# Risk Factors and outcomes associated with inappropriate empiric broad-spectrum antibiotic use in hospitalized patients with community-acquired pneumonia

**DOI:** 10.1017/ash.2023.258

**Published:** 2023-09-29

**Authors:** Tejal Gandhi, Lindsay Petty, Valerie Vaughn, Anurag Malani, David Ratz, Tawny Czilok, Jennifer Horowitz, Elizabeth McLaughlin, Lisa Dumkow, Stephanie Burdick, Danielle Osterholzer, Mariam Younas, Steven Bernstein, Scott Flanders

## Abstract

**Background:** Inappropriate broad-spectrum antibiotic use targeting methicillin-resistant *Staphylococcus aureus* (MRSA) and *Pseudomonas aeruginosa* can result in increased adverse events, antibiotic resistance, and *Clostridioides difficile* infection. In 2019, revised ATS/IDSA community-acquired pneumonia (CAP) guidelines removed healthcare-associated pneumonia (HCAP) as a clinical entity and modified patient factors warranting empiric broad-spectrum antibiotic (BSA) use. As a result, most patients hospitalized with CAP should receive empiric antibiotics targeting standard CAP pathogens. Based on revised guidelines, we evaluated predictors and outcomes associated with inappropriate BSA use among hospitalized patients with CAP. **Methods:** Between November 2019 and July 2022, trained abstractors collected data on non-ICU adult medical patients admitted with CAP at 67 Michigan hospitals who received either an inappropriate empiric BSA on hospital day 1 or 2 or a standard CAP regimen. Inappropriate empiric BSA use was defined as use of an anti-MRSA or anti-pseudomonal antibiotic in a patient eligible for standard CAP coverage per IDSA guidelines. Patients with immune compromise, moderate or severe chronic obstructive pulmonary disease (COPD), pulmonary complication, or guideline-concordant treatment with BSA were excluded. Data collected included comorbidities, antibiotic use and hospitalizations in the preceding 90 days, cultures in the preceding year, signs or symptoms of pneumonia, hospital characteristics, and 30-day postdischarge patient outcomes. Data were collected through chart review and patient phone calls. Predictors of inappropriate empiric BSA were evaluated using logistic general estimating equation (GEE) models, accounting for hospital-level clustering. We assessed the effect of inappropriate empiric BSA (vs standard CAP therapy) on 30-day patient outcomes using logistic GEE models controlling for predictors associated with the outcome and probability of treatment. **Results:** Of 8,286 included patients with CAP, 2,215 (26.7%) were empirically treated with inappropriate BSA. The median BSA treatment was 3 days (IQR, 2.5). After adjustments, factors associated with inappropriate empiric BSA treatment included hospitalization or treatment with high-risk antibiotics in preceding 90 days, transfer from a postacute care facility, hemodialysis, support with ≥3 L supplemental oxygen, severe sepsis, leukocytosis, and higher pneumonia severity index (Fig. 1). After adjustments, patients with inappropriate empiric BSA treatment had higher readmissions 30 days after discharge, more transfers to the intensive care unit, more antibiotic-associated adverse events, and longer hospitalizations (Fig. 2). **Conclusions:** Patients hospitalized with CAP often received inappropriate BSA as empiric coverage, and this inappropriate antibiotic selection was associated with worse patient outcomes. To improve patient outcomes, stewardship efforts should focus on reducing inappropriate BSA use in patients hospitalized for CAP with historic HCAP risk factors or severe CAP without other guideline-directed indications for BSA.

**Figure:**
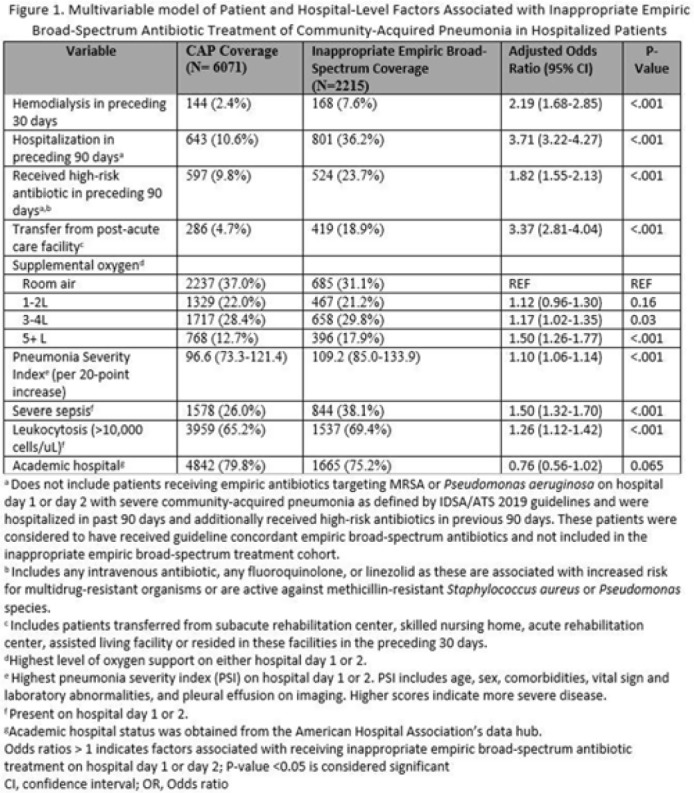

**Figure:**
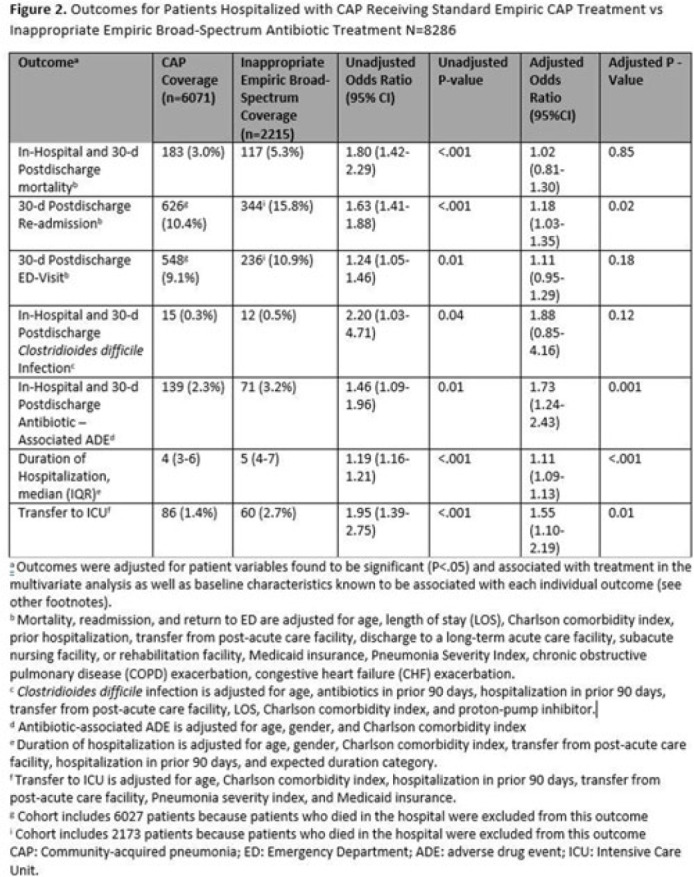

**Financial support.** H.M.S. initiative is underwritten by Blue Cross and Blue Shield of Michigan.

**Disclosures:** None

